# One-Step Femtosecond Laser Stealth Dicing of Quartz

**DOI:** 10.3390/mi11030327

**Published:** 2020-03-22

**Authors:** Caterina Gaudiuso, Annalisa Volpe, Antonio Ancona

**Affiliations:** 1 CNR-IFN UOS BARI, Via Amendola 173, 70126 Bari, Italy; antonio.ancona@uniba.it; 2 Dipartimento Interateneo di Fisica, Università degli Studi di Bari, 70125 Bari, Italy; annalisa.volpe@uniba.it

**Keywords:** ultrashort laser pulses, heat accumulation, transparent materials, quartz, stealth dicing

## Abstract

We report on a one-step method for cutting 250-µm-thick quartz plates using highly focused ultrashort laser pulses with a duration of 200 fs and a wavelength of 1030 nm. We show that the repetition rate, the scan speed, the pulse overlap and the pulse energy directly influence the cutting process and quality. Therefore, a suitable choice of these parameters was necessary to get single-pass stealth dicing with neat and flat cut edges. The mechanism behind the stealth dicing process was ascribed to tensile stresses generated by the relaxation of the compressive stresses originated in the laser beam focal volume during irradiation in the bulk material. Such stresses produced micro-fractures whose controlled propagation along the laser beam path led to cutting of the samples.

## 1. Introduction

Transparent materials are used in an increasing number of applications, ranging from microelectronics [1], to microfluidics [2,3] and optoelectronics [4]. In particular, glass, quartz and sapphire, due to their broad spectral band of light transmission, hardness and scratch-resistance, are good candidates for display components of portable mobile electronics, as light emitting diode (LED) substrates, protection mirrors of mobile phone cameras, smartwatches, etc. Despite their growing use in consumer electronics products, cutting these materials is still a challenging task, due to their brittleness. Obtaining a high-quality cut edge completely free from micro-cracks or chips is not easy, particularly when employing thinner substrates. The state of the art cutting technologies most widely used include traditional mechanical cutting, chemical etching, electrochemical machining and laser-based methods.

Traditional machining methods are mainly based on diamond cutting [4]. Here, the material is first marked and scribed with a diamond tool and then an external force is applied to break the substrate along the scribing path [5]. Unfortunately, with this method, at high cutting speeds the diamond blades can generate chipping and cracks, which compromise the quality of the cutting edges, reducing the resistance of the materials by up to 60% in the case of glass [6]. Moreover, tool wear affects the repeatability and the efficiency of the dicing process [7], and additional grinding and polishing steps are needed to achieve the required smooth finish.

Another method for cutting transparent materials is based on the selective chemical etching of laser modifications inside the volume [8]. The main advantage of this technique, besides the lack of debris, is the possibility of designing complex shapes. However, several modifications are required along the entire thickness of the substrate, thus limiting the processing speed. Furthermore, hydrofluoric acid (HF) is the only etchant which attacks amorphous SiO_2_, quartz or glasses at significantly high etching rates [9]. Unfortunately, due to its high toxicity and corrosiveness, when hydrogen fluoride (HF) is used for large scale productions, many countries require very strict safety regulations, see e.g., [10,11].

Electrochemical methods for machining quartz were reported by several authors [12,13,14,15]. Jain et al. [12] exploited electrochemical spark machining (ECSM) showing that, depending on the polarity and the applied voltage, a cut kerf ranging approximately from 0.5 to 0.9 mm and a surface roughness from 3 to 14 µm were obtained on a 2 mm-thick quartz sample. Wang et al. [15] demonstrated the shape cutting of quartz glass by wire electrochemical discharge machining (WECDM), but with this technique, significant bulges were generated on the edge of the cut circle.

In laser processing methods, the energy of a focused laser beam is exploited to modify the substrate from its surface or in the bulk, thus leading to its separation. Laser-based techniques are making huge advances in the field of cutting transparent materials, as they have numerous advantages over mechanical methods. Being non-contact, laser processes avoid any effect due to tool wear and mechanical stress, achieving high quality and precision of the cut at reasonable costs. Furthermore, the use of the laser prevents any contamination of the materials being processed. However, the process parameters, and in particular the wavelength of the laser source and its pulse width, must be carefully selected and adjusted to obtain the desired results and cutting-edge quality [16].

One of the first laser-based methods was introduced by Garibotti in 1963 [17] and consists in laser scribing and dicing. It is a two-step process: the laser is focused on the surface of the workpiece to generate a groove, which is subsequently fractured by applying a tensile stress. The mechanical stress that initiates the cutting process can be generated either by an external mechanical force or by a rapid heating-cooling cycle [10]. Serdyukov et al. [18] reported on numerical simulation about the controlled thermal cleavage of crystalline quartz, produced by laser heating and exposure to coolant. The cut was ascribed to the thermoelastic stress produced in the quartz crystals, due to the anisotropy of the thermal conductivity and thermal expansion. Such interpretation was confirmed through experiments performed using a CO_2_ laser, which led to the successful cut of quartz crystal, though with the presence of evident chips and micro-cracks.

In some cases, the laser irradiation itself provides enough thermal stress to cause the cutting of the sample. Such cutting process is referred to as thermal cleavage [19]. The focused laser spot acts, indeed, as a localized heat source generating a thermo-mechanical compressive stress, whose relaxation causes the material to separate along the laser scanning path [20]. The fracture mechanism is similar to a crack extension, which can deviate from the desired path, especially for fast cutting speeds and long cutting lengths [11]. After being optimized in order to control the path of the stress-induced fracture, such a technique has been successfully applied to cut various materials, including silicon [21], alumina ceramics [22] and glass [23]. Xu et al. [19] demonstrated the laser thermal cleavage of sapphire substrate wafers using a CO_2_ laser source. Here, a groove was engraved by laser ablation on the substrate surface. Laser irradiation caused localized and rapid heating and cooling, which led to the generation of local micro-cracks that propagated along preferential directions, with consequent cleaving and cutting of the substrate. Although attractive, this experimental procedure is very complex, since it requires cooling of the substrate and accurate alignment of the laser engrave to achieve the desired cutting path. Moreover, a protective layer must be applied to avoid surface contamination by redeposited laser ablation debris. An indirect, two steps thermal cleavage process has been developed by Choi et al. [16] for cutting glass, using a near-infrared (NIR) nanosecond laser. In this case, a laser-induced plasma plume was produced by irradiating a sacrificial absorbing layer, positioned a few hundred microns far from the glass target. Such a plume allowed the improvement of the absorption of laser energy by the target substrate, thus achieving localized and rapid heating and cooling. By delivering the appropriate amount of laser energy generating the plasma plume, controlled local microcracks were induced, which resulted in the cutting of the glass target.

A different approach was used by Russ et al. for cutting thin and ultra-thin glass plates (i.e., a thickness of a few hundred micrometers). Using ultrashort laser pulses, they ablated, layer-by-layer, the entire thickness of the substrate along the desired contour path [24]. This approach does not require applying any tensile stress and is suitable for any geometry, however it does not produce perpendicular cutting edges, has limited processing speed and produces a large amount of ablation debris [25]. Vanagas et al. used an analogous approach for the cutting of quartz and borosilicate cover glass samples, using 150 fs laser pulses at the wavelength of 800 m and repetition rate of 1 kHz. They demonstrated the feasibility of the laser cutting of quartz, although the processing speed was 200 µm/s and the laser-induced damage on the rear surface, caused by the multiple overlapped scanned paths, was quite extended [26].

Stealth dicing, instead, consists of focusing the laser beam inside the bulk material, transparent to the laser wavelength, and moving it along the desired path that acts as the initial division line, when an external tensile stress is subsequently applied. The process is ablation-free, does not generate any debris, and is extremely fast. The successful stealth dicing of thin sapphire wafers (350 μm) has been demonstrated using a fs-laser in the NIR wavelengths focused through a microscope objective [1]. However, single-focus stealth dicing laser processing does not always ensure a precise control of the taper, thus compromising the quality of the cutting edge. The laser cutting of thin borosilicate glass slides using a commercial fs-laser has been tested [27], finding that the fine cut can be successfully obtained by carefully adjusting the scan speed, in order to trigger the formation of micro-cracks at the exit of the laser beam from the sample. The generation of such microcracks was ascribed to the damage induced by filamentation and the consequent mechanical stress built-up in the material. Moreover, many studies have explored multifocal laser processing, mainly using a combination of diffractive optical elements (i.e., Fresnel lens) and Bessel beams [22,23]. Tsai et al. [28] demonstrated the cutting of a thin glass with a thickness of 100 µm through modification in the bulk volume, using a femtosecond laser Bessel beam and applying a breaking stress. A smooth cut edge with chipping <1 µm was obtained. Dudutis et al. [29] proved the possibility to use a picosecond laser for glass dicing using an axicon-generated asymmetrical Bessel beam. A tilt movement was applied to the axicon holder in order to add an astigmatic aberration, producing a Bessel beam with controlled asymmetry. This solution allowed the achievement of 2.8 times faster dicing speed and the lower propagation of cracks into the bulk material, compared to dicing with a symmetrical Bessel beam. The stealth dicing of sapphire using ultrashort pulsed Bessel beams has also been demonstrated by Lopez et al. [30], but achieving a sidewall roughness of around 2 μm.

In this work, we investigate the cutting of quartz by ultrashort laser pulses. Quartz is a material of relevant interest in many fields like e.g., optoelectronics, fibre-technology and photoacoustics [31,32], thanks to its high optical transmission in a broad range from ultraviolet (UV) to mid- infrared (MIR), unique thermal and electrical properties, besides an excellent chemical resistance. The high precision cutting of quartz is crucial for many applications that require the fabrication of miniaturized quartz-based devices. The traditional method for cutting quartz is based on lithography followed by wet etching, using highly toxic agents like ammonium bifluoride [17] or HF acid [8]. The laser-based cutting method proposed in this work is a clean, single pass stealth dicing process, which does not involve any chemical agent or external tensile stress. The dependence of the main laser process parameters as the repetition rate, the pulse overlap and the pulse energy on the cutting efficiency were experimentally investigated. The quality of the cut edge has been thoroughly analyzed by optical microscopy. Optical profilometry has been exploited to evaluate the roughness of the cut edge and the flatness of the final cuts.

## 2. Materials and Methods

**Figure 1:**
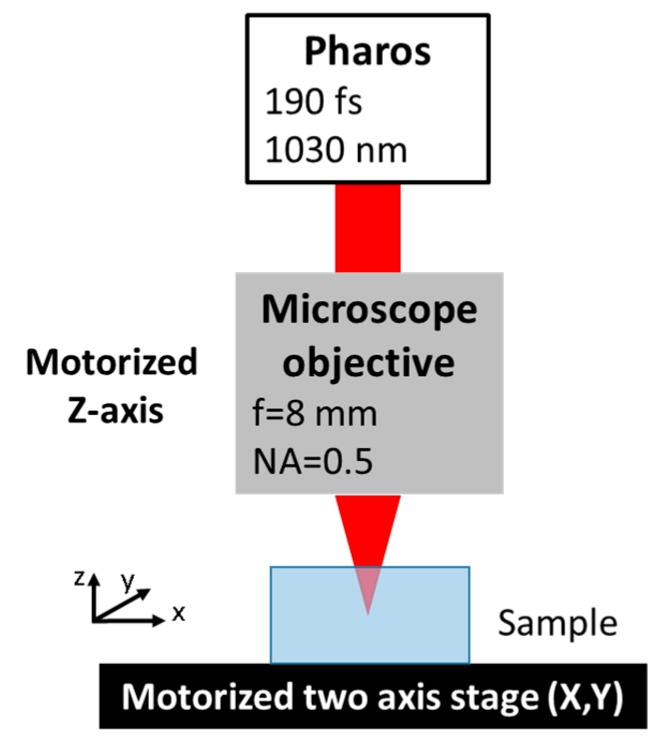
Experimental set-up used for the cutting experiments.

The set-up used for these experiments is shown in Figure 1. The laser source was the Pharos SP 1.5 from Light Conversion, providing 200 fs pulses with variable repetition rate from a single pulse to 1 MHz. The almost diffraction limited laser beam (M^2^<1.3) was characterized by a central wavelength of 1030 nm and had a maximum average power of 6 W and maximum pulse energy *E_p_* of 1.5 mJ.

The linearly polarized exit beam passed through a half-wave plate and a polarizer, which allowed one to tune the average power by rotating the half waveplate. Next, the laser beam was sent to a microscope objective with a focal length of 8 mm (the estimated focused spot diameter *d* in air was 1.3 µm) mounted on a PC controlled motorized axis (Aerotech, ANT130 LZS, Pittsburgh, PA, USA). This enabled the beam focus to be finely positioned in the bulk of the transparent samples that were moved on a XY plane, perpendicular to the beam axis by two Aerotech ABL1500 motorized stages with sub-micrometer resolution. As samples, 250 µm thick Z-cut quartz plates from Nano Quartz Wafer were used. In the present experimental conditions, self-focusing is likely experienced at a distance from the sample surface estimable as [33]:

(1) $$z_{\mathrm{sf}}=\frac{2n_{0}{\left(d/2\right)}^{2}}{\lambda }\frac{1}{\sqrt{P/P_{\mathrm{cr}}}}$$

where *n*_0_ is the linear refractive index and λ the wavelength, P is the applied peak power and *P*_cr_=11 MW is the critical power for having self-focusing, determined according to Eq. 7.1.1 in [33]. Considering that the applied peak power ranged from 75 MW to 175 MW, the calculated self-focusing distance ranged between 0.31 µm and 0.47 µm. Correspondingly, the spot radius at such distance was determined according to [34]:

(2) $$w_{\mathrm{sf}}^{2}\left(z_{\mathrm{sf}}\right)={\left(d/2\right)}^{2}\left[{\left(1+\frac{z_{\mathrm{sf}}}{R_{0}}\right)}^{2}+\frac{4\gamma }{k^{2}{\left(d/2\right)}^{4}}z_{\mathrm{sf}}^{2}\right]$$

where *R*_0_ is the curvature radius, *k* is the wave number in linear media and $\gamma =\left(1+\alpha^{-2}\right)\left(1-\frac{P}{P_{\mathrm{cr}}}\right)$, with *α* the degree of global coherence. In the present case, considering *α*→∞, the estimated beam spot radius due to self-focusing varied from 0.42 µm to 0.52 µm at the self-focusing distances, according to the applied peak power. However, it is important to highlight that the aberration occurring from focusing the laser beam deep inside the quartz sample could cause deviations from a diffraction-limited beam spot, by extending the focal region along the optical axis.

The translation speed *v* and the repetition rate *f* defined the average number of pulses pps (pulses per spot) impinging on the same focal area, calculated as pps = *w*_sf_*f/v.

The repetition rate, the scan speed and the pulse energy were varied and their influence on the cutting process was evaluated, in order to better understand the underlying physical mechanisms.

## 3. Results and Discussion

### 3.1. Influence of the Repetition Rate and pps

It was found that the repetition rate plays a fundamental role to get the laser-induced dicing of the quartz plates in a single step. Indeed, the repetition rate defines the time separation between two successive laser pulses and together with the travel speed and the pulse energy, determines the amount of laser energy and heat released in a given material volume per unit time.

Using a repetition rate of 25 kHz, the single-step cutting of quartz was never achieved regardless the travel speed and, thus, the number of pps. At higher repetition rates, stealth dicing of the plates occurred with quite different cutting edge qualities, depending on the pulse energy and the number of pps.

**Figure 2:**
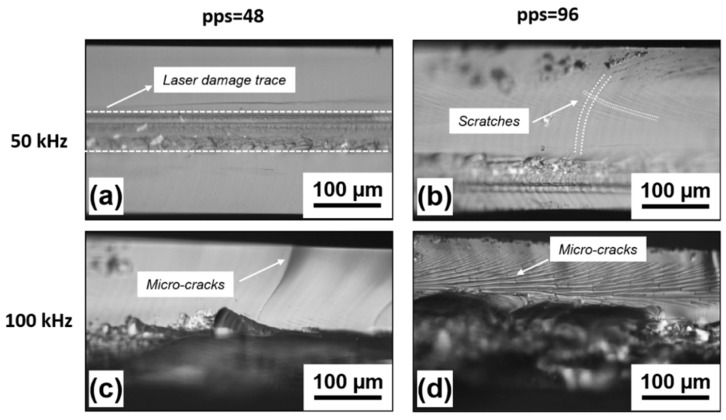
Optical microscope images of the cut edges obtained with a fixed pulse energy *E_p_*=20 µJ and different repetition rates of 50 kHz and 100 kHz and pulse overlap pps= 48 and 96 (**a**–**d**).

In Figure 2, the optical microscope images of the cut edges obtained on quartz at 50 kHz and 100 kHz, with a pulse energy of 20 µJ and pps of 48 and 96, respectively, are shown. All the cuts exhibit a damaged area beneath the surface, in the region where the laser focus was placed. The areas above and below appear to be rather clean.

Several physical mechanisms, generally taking place when irradiating a transparent material with intense ultrashort laser pulses, might have concurred to get the single-step stealth dicing of quartz. Besides self-focusing, also aberration from focusing deep inside the bulk quartz and filamentation typically occur at the peak power levels used in our experimental conditions [35]. However, the laser damaged area is quite confined inside the bulk material. Therefore, beam filamentation unlikely occurred along the whole quartz thickness. It is much more plausible that the physical mechanism originating the single pass stealth dicing is an accumulation of laser-induced stresses. In fact, in agreement with [36], it can be estimated that the peak temperature reached by the quartz lattice after each laser pulse is around 10^3^ K. At such a high temperature, a transient tensile stress is generated, whose magnitude can be estimated by the following formula [16]:

(3) $$\sigma =\frac{E\alpha \Delta T}{1-\upsilon }$$

where *E* is the Young modulus, *α* is the coefficient of thermal expansion, ∆T is the temperature increase due to irradiation, and ν is the Poisson’s number. The magnitude of such stress, which has been calculated taking into account the specific experimental conditions, is of the order of some hundreds of MPa. Literature reports a formation of cracks when the stress exceeds 1 GPa [37]. Therefore, the generation of a crack is not expected after each single pulse. However, since the single-pulse laser-generated mechanical stress lasts longer than 10 µs [38], if a second pulse arrives before such stress is released, then the two stresses accumulate. Pulse after pulse, this stress accumulation mechanism leads to the generation of cracks. The joining and propagation of cracks following the laser path and throughout the entire thickness of the quartz plate originates the single step stealth dicing process.

This explanation justifies the different results obtained at the three investigated values of repetition rate. In fact, at 25 kHz, the time delay between subsequent pulses is much longer than the relaxation time of each laser induced stress. Therefore, the stresses of following pulses do not overlap, and dicing of the sample does not take place. As far as the repetition rate increases, the pulse-to-pulse time interval shortens and the laser-induced stresses start to accumulate, finally resulting in the formation of cracks and dicing. At a repetition rate of 50 kHz, the time delay between consecutive pulses is half of the previous case and the self-induced stealth dicing of the samples was successfully obtained, with overall acceptable quality of the cut edges, especially for pps = 48.

In this case, the area modified by the laser interaction is well confined in the bulk volume and can be easily recognized, as shown in Figure 2a. Here, the average areal roughness Sa was found to be around 1 µm. No significant collateral damage is noticed above or below the laser modification trace and a much lower surface roughness of around 0.05 µm was found. By increasing the number of pps to 96, the quality of the cut edge is still acceptable, but some scratches begin to be noticed above and below the laser modified volume, see Figure 2b.

At an even higher repetition rate of 100 kHz, the laser induced crack propagation mechanism is no longer under control, owing to the excessive thermal load released into the focal volume [39]. This causes significant collateral damage around the laser absorption area, with unacceptable quality of the cut edges, as shown in Figure 2c,d. In particular, in the top part of the cut edge, long cracks propagating all the way towards the surface can be noticed at the lower number of pps = 48. For higher pps = 96, a large number of very dense erosion lines, accompanied by microcracks bridging them, appear in the upper part of the cut edge. For both investigated pps values, at 100 kHz of repetition rate, the lower part of the cut edge is completely destroyed, with big chips of quartz that have detached from the bottom.

**Figure 3:**
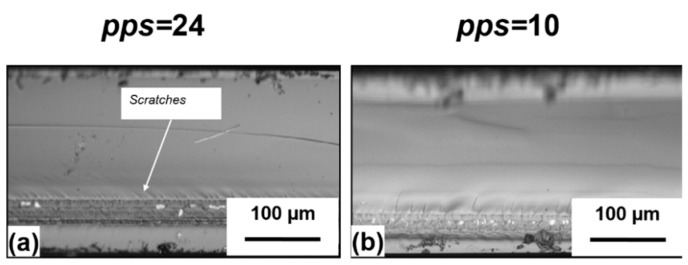
Optical microscope images of the cut edges obtained with pps = 24 and 10, at 50 kHz repetition rate and a pulse energy of 20 µJ. The reduction of the pps from 24 to 10 causes the cut edge not to be perpendicular to the target surface. In (**a**), the image is almost entirely on focus. In (**b**), the part of the cutting edge above the laser damage trace is out of focus, thus indicating a different height with respect to the part below.

The role of the overlap between pulses has been further investigated by performing additional experiments at the repetition rate of 50 kHz, keeping the same pulse energy of 20 µJ and reducing the number of pps to 24 and 10, respectively. Even in these two cases, the single step laser stealth dicing of the quartz samples was achieved. The corresponding optical microscope images of the cut edges are shown in Figure 3. As for pps = 48, the average areal roughness Sa of the laser modified zone was equal to 1 µm for both samples, while the surrounding area showed a surface roughness around 0.05 µm. However, compared to the case of pps = 48 shown in Figure 2a, where the cut edge was almost perfectly flat without any evident imperfection, a reduction of the number of pps has not led to further improvement of the cut quality. Unexpectedly, some small scratches or micro-cracks appear close to the laser modified area, suggesting that besides the repetition rate, the number of pps must also be selected within an ideal range to obtain a clean single step cut. On the other hand, a slight decrease of the laser damaged area depth, from approximately 50 µm to 35 µm, is noticed when decreasing the pps from 48 to 10. This is ascribable to the well-known incubation effect [40]. As the number of impinging pulses increases, the damage threshold decreases through the creation of point defects. Those defects enhance absorption of subsequent pulses, thus improving the coupling of laser energy into the lattice [40]. As a consequence, the dimensions of the laser-modified area increase with the number of pps. In addition, an increase of the pps leads to an increment of the accumulated fluence (defined as the pps multiplied by the fluence of the single pulse), which may change the morphology of the laser damage trace [41].

### 3.2. Influence of the Pulse Energy

The influence of the pulse energy on the overall cutting mechanism and the cut edge quality has been investigated by carrying out experiments at 50 kHz of repetition rate, 1 mm/s of translation speed and varying the pulse energy from 15 to 40 µJ, with increments of 5 µJ. Indeed, below 15 µJ, the pulse energy was too low to get the single step cutting of the samples, thus indicating that it is a threshold process.

**Figure 4:**
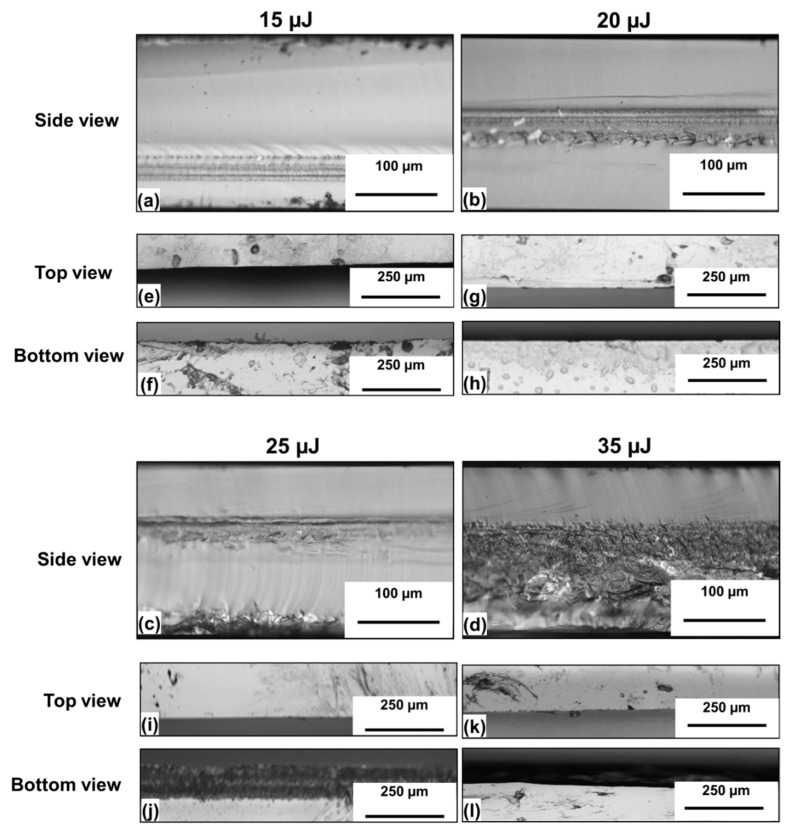
(**a**–**d**) Side view of the cut edges obtained at 50 kHz, at the scan speed of 1 mm/s and four different pulse energies. The top (**e**,**g**,**i**,**k**) and the bottom (**f**,**h**,**j**,**l**) views are also shown.

**Figure 5:**
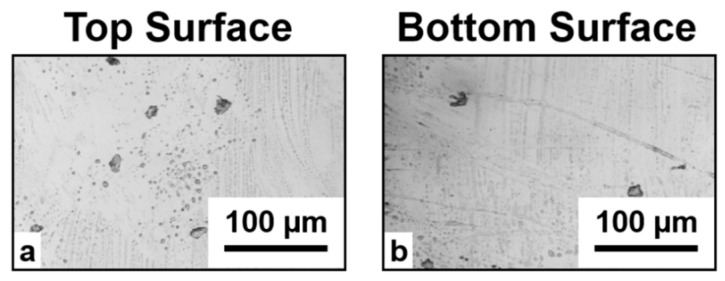
Top (**a**) and bottom (**b**) surface of the target before laser irradiation.

The corresponding cut edges are shown in Figure 4, where the top and bottom views are also presented. For Ep = 15 µJ (Figure 4a) a single step stealth dicing cut with acceptable quality of the cut edge was obtained, except for some small imperfections on the top of the laser modified area, which had a depth of 45 µm. The best cut quality was obtained at 20 µJ, where a clean cleavage is observed, with a 50-µm-deep laser modified zone buried inside the sample thickness. The cut is straight with well-defined edges following the laser path, and very few surface defects, as can be noticed from the top and bottom view of the sample, unlike the case reported by Vanagas et al. [26], where a damage area on the sample rear surface of approximately 500 µm wide was observed. A comparison with the top and bottom view of the same sample before irradiation (Figure 5) does not show any significant difference. Therefore, it can be excluded that when focusing the laser beam inside the bulk material surface, defects are generated on the top and the bottom of the sample.

As the pulse energy was increased to above 25 µJ, a clear ablation occurred at the bottom of the sample. At an even higher value of pulse energy of 35 µJ, the damaged region merged with the laser trace, reaching an extension of almost 160 µm and resulting in cuts of poor quality.

**Figure 6:**
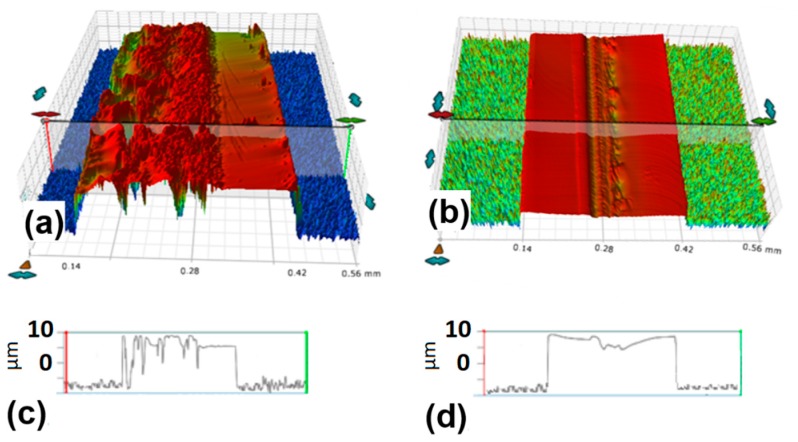
Three-dimensional profiles and line profiles of the cuts obtained at 50 kHz, scan speed of 1 mm/s and pulse energy of (**a**,**c**) 35 µJ and (**b**,**d**) 20 µJ, respectively.

In Figure 6, the 3D and line profiles of the cut edges obtained for 50 kHz of repetition rate, 1 mm/s of scan speed and pulse energies of 35 µJ and 20 µJ, respectively, are shown.

At the higher pulse, energy evident laser-induced damage is present, as also clearly visible in Figure 4d,l. The cut edge obtained with the lower pulse energy has a significantly smoother surface, except for slight erosion lines on the right side and a small depression, only a few micrometers high, positioned at the laser trace in the middle of the cut edge.

## 4. Conclusions

We performed a systematic study on the stealth dicing process of quartz plates using ultrashort laser pulses. The influence of the main laser parameters as the repetition rate, the pulse energy, the translation speed and, as a consequence, the pulses overlap, on the cut efficiency and quality has been thoroughly investigated.

We have found that the single pass self-induced laser cutting of quartz is possible within the range of plate thickness explored in this work (250 µm). The physical mechanisms leading to the cleavage have been ascribed to the accumulation, pulse after pulse, of tensile stresses, generated by the rapid increase of temperature experienced in the focal volume after the nonlinear absorption of laser energy. Such stresses cause micro-fractures that produce the cut, while propagating along the laser path and throughout the entire thickness of the plate.

The repetition rate has been found to be a key parameter to generate a controlled and clean cut, since this parameter defines the time delay between successive pulses and thus the amount of stress accumulated in the focal volume. In fact, a repetition rate of 25 kHz was found to be too low to initiate the crack, while at 100 kHz, the bottom part of the plates was completely disrupted, due to the excessive laser induced stress. The optimal value of repetition rate, which has allowed obtaining a neat cut without any significant damage above or below the laser modified zone, was 50 kHz.

Increasing the overlap between pulses or the pulse energy and keeping the same repetition rate resulted in reducing the quality of the final cut, with the appearance of erosion lines and chipping around the laser modified zone. This indicates that a higher energy load causes, once again, excessive laser induced stresses and/or the initiation of ablation from the top or the bottom surface, thus making the stealth dicing process unstable.
